# Risk factors for incident falls in older men and women: the English longitudinal study of ageing

**DOI:** 10.1186/s12877-018-0806-3

**Published:** 2018-05-16

**Authors:** Catharine R. Gale, Leo D. Westbury, Cyrus Cooper, Elaine M. Dennison

**Affiliations:** 1 MRC Lifecourse Epidemiology Unit, University of Southampton, Southampton General Hospital, Southampton, SO16 6YD UK; 2 Centre for Cognitive Ageing & Cognitive Epidemiology, Department of Psychology, University of Edinburgh, Edinburgh, UK

**Keywords:** Falls, Gender, Risk factors

## Abstract

**Background:**

Falls are a major cause of disability and death in older people, particularly women. Cross-sectional surveys suggest that some risk factors associated with a history of falls may be sex-specific, but whether risk factors for incident falls differ between the sexes is unclear. We investigated whether risk factors for incident falls differ between men and women.

**Methods:**

Participants were 3298 people aged ≥60 who took part in the Waves 4–6 surveys of the English Longitudinal Study of Ageing. At Wave 4, they provided information about sociodemographic, lifestyle, behavioural and medical factors and had their physical and cognitive function assessed. Data on incident falls during the four-year follow-up period was collected from them at Waves 5 and 6. Poisson regression with robust variance estimation was used to derive relative risks (RR) for the association between baseline characteristics and incident falls.

**Results:**

In multivariable-adjusted models that also controlled for history of falls, older age was the only factor associated with increased risk of incident falls in both sexes. Some factors were only predictive of falls in one sex, namely more depressive symptoms (RR (95% CI) 1.03 (1.01,1.06)), incontinence (1.12 (1.00,1.24)) and never having married in women (1.26 (1.03,1.53)), and greater comorbidity (1.04 (1.00,1.08)), higher levels of pain (1.10 (1.04,1.17) and poorer balance, as indicated by inability to attempt a full-tandem stand, (1.23 (1.04,1.47)) in men. Of these, only the relationships between pain, balance and comorbidity and falls risk differed significantly by sex.

**Conclusions:**

There were some differences between the sexes in risk factors for incident falls. Our observation that associations between pain, balance and comorbidity and incident falls risk varied by sex needs further investigation in other cohorts.

## Background

Falls are a major cause of unintentional injury worldwide [1]. They are the most common type of accident in people aged 65 or older, and can result in death, hospitalisation, disability, loss of independence, and also fear of falling which can lead to activity restriction and decline in physical function [2, 3]. The economic costs of falls as regards medical and social care are substantial [1, 4–6].

Women have a higher risk of falls than men [7–9]. Yet although there have been many investigations into risk factors for falls, very few have examined men and women separately. In two large nationally-representative surveys in Canada and England, there was evidence of differences between the sexes in some risk factors for falls [9, 10]. A limitation of these studies is that both were cross-sectional so it was not always possible to be certain about the direction of effect. To determine whether falls prevention programmes should be designed with gender in mind, it is important to have longitudinal evidence of whether the factors that predict incident falls differ between the sexes.

In the current prospective study, we investigated risk factors for incident falls over an average period of 4 years in men and women separately using data from the English Longitudinal Study of Ageing (ELSA).

## Methods

### Participants

The original ELSA sample consisted of individuals at least 50 years of age who had taken part in the Health Survey for England in 1998, 1999 or 2001 [11]. The sample was selected by postcode sector and stratified by both health authority and the percentage of households belonging to non-manual socioeconomic classes. The original survey occurred in 2002–3 and later waves of data collection have taken place every 2 years. Every 4 years, participants are invited to have a visit from a nurse during which physical function is measured. To ensure the representation of people aged 50–75 was maintained, additional samples from the Health Survey for England were added at Waves 3 and 4. For the current study, we used data from Waves 4, 5 and 6 [12].

### Measures

#### Falls

At Wave 4, participants aged 60 years or over were asked “Have you fallen down in the last year (for any reason)?” This question was used to derive a variable for previous falls. At Waves 5 and 6, participants aged 60 or over who had taken part in previous sweeps of the study were asked “Since we last talked to you on [date of last interview inserted] have you fallen down (for any reason)?” These questions were used to derive a variable for the study outcome, incident falls during the 4 year period between Waves 4 and 6.

#### Independent variables

Potential risk factors for falls were selected on the basis of previous literature [9, 10, 13–20]. Sociodemographic characteristics consisted of age, sex, marital status (married/cohabiting, widowed/divorced/separated or single) and socioeconomic position. We used total household wealth (including savings and investments, value of property or business assets, net of debt, excluding pension assets) as our measure of socioeconomic position. Lifestyle factors consisted of body mass index (BMI), smoking status, alcohol consumption and physical activity. Clinical characteristics consisted of morbidity burden (indicated by number of diagnosed conditions), physical frailty as defined by the Fried phenotype [21], symptoms of depression and reported problems regarding hearing, eyesight, frequent pain, or incontinence. Measures relating to physical function and cognitive ability consisted of balance, lung function and general cognitive function. We did not include walking speed or grip strength as independent variables because they are both used to define the Fried phenotype and are highly correlated with it.

BMI was derived from weight and height measured by a nurse. Participants provided information on smoking status, categorized as current smoker, ex-smoker or never smoked. They responded to a question about how much physical activity their job involved (if they were employed) and to three questions about mild, moderate or vigorous physical activity in their everyday life. A summary variable on physical activity was derived from this information, categorizing participants’ degree of activity as sedentary, low, moderate or high. This is very similar to the classification used in the Allied Dunbar Survey of Fitness [22]. Participants supplied data on how often they drank alcohol in the last year in five categories, ranging from ‘almost every day’ to ‘not at all.’

Participants were asked to indicate whether a doctor had told them that they had any of the following illnesses: hypertension or high blood pressure, angina, heart attack or myocardial infarction, congestive heart failure, diabetes, stroke, chronic lung disease, asthma, arthritis, Parkinson’s disease, Alzheimer’s disease, dementia or memory impairment, psychiatric or emotional problems, cancer or osteoporosis. We added the number of illnesses present as a marker of morbidity burden. This simple measure is the most common way of ascertaining morbidity burden [23], and has been shown to be almost as effective at predicting mortality and health care costs as more complex methods [24].

Participants completed the 8-item version of the Centre for Epidemiologic Studies Depression Scale (CES-D) which measures symptoms of depression [25].

During a home visit a nurse used standardized protocols to assess balance, grip strength and lung function, as described previously [9]. The balance assessment involved the participants completing up to three stands, a side-by-side (stand with feet together, side by side); a semi-tandem (stand with the side of the heel of 1 foot touching the big toe of the other foot); and a full-tandem (stand with the heel of 1 foot in front of and touching the toes of the other foot).Each of these was demonstrated by the nurse to the respondent beforehand. The first stand performed by the participants (provided it was judged safe for them to do so) was the side-by-side stand. Participants who were able to do this stand for 10 s were asked to do a semi-tandem stand for 10 s. If participants were able to hold the semi-tandem stand for the required time, they were asked to perform a full tandem stand. The time participants were asked to hold the full tandem stand was 30 s if they were ≤ 69 years and 10 s if they were ≥ 70. In the current study, we used data on the full tandem stand.

Grip strength of each hand was measured three times with a Gripometer. The maximum of these measurements was used. Lung function was assessed using a NDD Easy On Spirometer. The highest technically satisfactory measure of forced expiratory volume in 1 sec (FEV1) was used for analysis.

Usual walking speed was assessed by measuring the time taken to walk 8 ft, once the interviewer judged the participant could do this safely. They were allowed the use of walking aids. Participants who needed supporting by another person or who were thought to be likely to fall were not invited to do the test. The timed walk was repeated and the mean of the two measurements was used for analysis.

Frailty or pre-frailty according to the Fried phenotype is defined by the presence of 3 or more, or 1 or 2 respectively, of the following components: unintentional weight loss, weakness, self-reported exhaustion, slow walking speed and low physical activity [21]. These components were operationalized as follows. Weight loss was considered present if participants had lost at least 10% of their weight since Wave 2 or their BMI was presently less than 18.5 kg/m^2^. Weakness was considered present if participants had a grip strength that was in the lowest 20% of the distribution once we had adjusted for sex and BMI. Exhaustion was defined as a positive response to either of the CES-D items ‘Felt that everything I did was an effort in the last week’ or ‘Could not get going in the last week’. Slow walking speed was considered present if participants had a walking speed that was in the lowest 20% of the distribution once we had adjusted for sex and height; (those unable to do the timed walk due to concerns about safety, health problems or lack of a walking aid were counted as having slow walking speed). Low physical activity was defined as physical activity in the lowest sex-specific 20% of the distribution.

Incontinence was assessed by a question asking whether they had lost any amount of urine beyond their control in the last 12 months. Pain was assessed by asking participants if they were often troubled by pain. Those who answered ‘yes’ were asked to indicate whether the pain was mild, moderate or severe. Participants were asked to rate their hearing (with a hearing aid if used) as excellent, very good, good, fair or poor. Participants were asked to rate their eyesight (with glasses if used) as excellent, very good, fair, poor or registered or legally blind.

Participants took tests of cognitive function as follows. Verbal memory was assessed by asking participants to recall immediately 10 nouns that were presented to them aurally. Prospective memory was assessed by asking participants to remember to carry out a particular task later in the interview. Attention was assessed using a timed letter cancellation task (crossing out as many target letters as possible on a page containing letters in a grid). Executive function was assessed by testing participants’ verbal fluency. For this they had 60 s to name as many animals as possible. We subjected the scores from these four tests to principal components analysis and extracted the first unrotated principal component which accounted for 46% of the variance. This provided a standardized measure of general cognitive ability.

#### Analytical sample

In total, 5896 core participants aged 60 or over took part in the nurse visit at Wave 4; of these participants, 4735 (80%) participated at Waves 5 and 6. Of these 4735 participants, 3298 (70%) had complete data on variables of interest and were included in the analysis. All of them were interviewed in person rather than by proxy.

#### Statistical methods

Data were described using summary statistics. Poisson regression with robust variance estimation was used to derive relative risks for the association between participant characteristics at baseline, Wave 4, and the risk of falling between Waves 4 and 6. Initial regression models examined each characteristic separately and adjusted for history of falls only. Variables that were associated with falling (*p* < 0.2) were then included in sex-specific mutually-adjusted models as in previous studies [9, 10]. Analyses were performed on men and women separately. To ensure effect sizes were comparable, sex-specific SD scores were derived for continuous characteristics (BMI was log-transformed prior to standardising). Statistical analysis was performed using Stata, release 14 [26].

## Results

Of the 3298 people in the study, 633 (41.8%) men and 863 (48.4%) women experienced an incident fall between Waves 4 and 6. The number of men and women at baseline who experienced a fall in the previous year was 319 (21.1%) and 487 (27.3%) respectively. The relative risk of an incident fall among those with a previous fall before wave 4, compared to those without, was 1.67 (95%CI 1.55, 1.79) after adjustment for age and sex.

### Summary statistics and minimally-adjusted associations

Summary statistics for sociodemographic and lifestyle characteristics at baseline (Wave 4) and the relative risks for incident falls between Waves 4 and 6 according to these characteristics are presented in Table 1. These relative risks have been adjusted only for history of previous falls. Among both sexes, risk of incident falls increased with age and was higher among those who were divorced, widowed or separated compared to those who were married or cohabiting. Household wealth and BMI were not associated with fall risk (*p* > 0.05). We carried out an additional analysis to check whether there was a non-linear relationship between BMI and fall risk, but there was no evidence of that in either sex. Among men only, lower levels of physical activity, lower alcohol consumption and being a current smoker were each associated (*p* < 0.05) with increased risk of incident falls. Among women only, those who had never married were at the highest risk of incident falls, compared to other relationship groups.

**Table 1:** Summary statistics for socio-demographic and lifestyle factors at Wave 4 and relative risks of incident falls between Waves 4 and 6 according to these characteristics among 1515 men and 1783 women aged 60 and over

Characteristic	Men			Women		
	*N*(%)	Relative risk (95% CI)	*P*-value	*N*(%)	Relative risk (95% CI)	*P*-value
Age (years)^a^	68.9 (6.8)	1.15 (1.10,1.21)	< 0.001	69 (7.1)	1.12 (1.07,1.17)	< 0.001
Marital status						
Married/cohabiting	1238 (81.7%)			1117 (62.6%)		
Divorced/widowed/separated	213 (14.1%)	1.19 (1.03,1.37)	0.058	603 (33.8%)	1.18 (1.07,1.30)	< 0.001
Never married	64 (4.2%)	1.06 (0.81,1.38)		63 (3.5%)	1.35 (1.10,1.64)	
Household wealth^b^						
Poorest quintile	169 (11.2%)	0.97 (0.93,1.01)	0.177	257 (14.4%)	0.99 (0.96,1.03)	0.744
Second	234 (15.4%)			329 (18.5%)		
Third	307 (20.3%)			392 (22%)		
Fourth	379 (25%)			393 (22%)		
Highest quintile	426 (28.1%)			412 (23.1%)		
BMI (kg/m^2^)^a^	28.2 (4.3)	1.02 (0.97,1.08)	0.453	28.3 (5.5)	1.04 (0.99,1.09)	0.106
Smoking status						
Never	458 (30.2%)	1.00	0.081	843 (47.3%)	1.00	0.509
Ex	917 (60.5%)	1.13 (0.98,1.29)		760 (42.6%)	1.03 (0.93,1.13)	
Current	140 (9.2%)	1.24 (1.01,1.52)		180 (10.1%)	0.93 (0.78,1.11)	
Physical activity^b^						
Sedentary	49 (3.2%)	0.86 (0.80,0.92)	< 0.001	71 (4%)	0.97 (0.92,1.03)	0.372
Low	253 (16.7%)			449 (25.2%)		
Moderate	844 (55.7%)			928 (52%)		
High	369 (24.4%)			335 (18.8%)		
Alcohol consumption^b^						
Not at all in past year	110 (7.3%)	0.94 (0.90,0.98)	0.009	214 (12%)	0.99 (0.96,1.03)	0.688
≥ 1/2 times per year	124 (8.2%)			391 (21.9%)		
1/2 times per month	143 (9.4%)			235 (13.2%)		
≥ 1/2 times per week	802 (52.9%)			701 (39.3%)		
Almost every day	336 (22.2%)			242 (13.6%)		

*P*-value for difference in risk of falling between categories shown for smoking and marital status

Poisson regression models with robust variance estimation were used to yield relative risks

Relative risk estimates were adjusted for previous falls before Wave 4

^a^Mean (SD) for summary statistics and relative risks correspond to SD increases

^b^Relative risk per higher category

Summary statistics for clinical, physical and cognitive characteristics at Wave 4 and the relative risks for falling between Waves 4 and 6 according to these characteristics are presented in Table 2. Among both men and women, the following characteristics were associated (*p* < 0.05) with increased risk of falls: increased comorbidity; poorer eyesight; incontinence; experiencing higher levels of pain; increased depression score; poorer performance regarding the balance and lung function tests; being frail or pre-frail; and poorer cognition. Poorer hearing was associated (*p* < 0.05) with increased risk of falling among men only.

**Table 2:** Summary statistics for clinical, physical and cognitive factors at Wave 4 and relative risks of incident falls between Waves 4 and 6 according to these characteristics among 1515 men and 1783 women aged 60 and over

Characteristic	Men			Women		
	*N* (%)	Relative risk (95% CI)	*P*-value	*N* (%)	Relative risk (95% CI)	*P*-value
Number of comorbidities^b^	1 (0, 2)	1.10 (1.06,1.14)	< 0.001	2 (1, 2)	1.07 (1.04,1.11)	< 0.001
Hearing^c^						
Excellent	195 (12.9%)	1.08 (1.02,1.13)	0.006	361 (20.2%)	1.05 (1.00,1.09)	0.054
Very good	369 (24.4%)			538 (30.2%)		
Good	551 (36.4%)			614 (34.4%)		
Fair	312 (20.6%)			224 (12.6%)		
Poor	88 (5.8%)			46 (2.6%)		
Eyesight^c^						
Excellent	255 (16.8%)	1.12 (1.06,1.19)	< 0.001	244 (13.7%)	1.08 (1.03,1.13)	0.002
Very good	558 (36.8%)			605 (33.9%)		
Good	547 (36.1%)			726 (40.7%)		
Fair	136 (9%)			157 (8.8%)		
Poor/blind	19 (1.3%)			51 (2.9%)		
Incontinence^d^	116 (7.7%)	1.29 (1.11,1.51)	0.001	391 (21.9%)	1.20 (1.09,1.33)	< 0.001
Troubled by pain^c^						
None	1010 (66.7%)	1.15 (1.09,1.21)	< 0.001	1013 (56.8%)	1.09 (1.04,1.13)	< 0.001
Mild	190 (12.5%)			193 (10.8%)		
Moderate	250 (16.5%)			430 (24.1%)		
Severe	65 (4.3%)			147 (8.2%)		
CES-Depression score^b^	0 (0, 1)	1.07 (1.04,1.10)	< 0.001	1 (0, 2)	1.05 (1.03,1.08)	< 0.001
Full-tandem stand						
≥ 10 s if aged ≥70/≥30 s if aged < 70	1266 (83.6%)	1.00	< 0.001	1324 (74.3%)	1.00	< 0.001
< 10 s if aged ≥70/< 30 s if aged < 70	172 (11.4%)	1.28 (1.09,1.49)		299 (16.8%)	1.16 (1.03,1.31)	
Not attempted	77 (5.1%)	1.58 (1.36,1.84)		160 (9.0%)	1.31 (1.15,1.49)	
Frailty status						
Not frail	889 (58.7%)	1.00	< 0.001	984 (55.2%)	1.00	< 0.001
Pre-frail	537 (35.4%)	1.27 (1.12,1.44)		641 (36%)	1.14 (1.03,1.26)	
Frail	89 (5.9%)	1.66 (1.42,1.94)		158 (8.9%)	1.35 (1.18,1.54)	
Forced expiratory volume (litres)^a^	2.8 (0.8)	0.91 (0.86,0.96)	0.001	1.9 (0.5)	0.93 (0.89,0.97)	0.001
Cognition^a^	−0.06 (0.89)	0.91 (0.86,0.96)	0.001	0.08 (0.88)	0.94 (0.90,0.98)	0.008

*P*-value for difference in risk of falling between categories shown for full-tandem stand and frailty status

Poisson regression models with robust variance estimation were used to yield relative risks

Relative risk estimates were adjusted for previous falls before Wave 4

^a^Mean (SD) for summary statistics and relative risks correspond to SD increases

^b^Median (lower quartile, upper quartile) for summary statistic and relative risks correspond to unit increases

^c^Relative risk per higher category

^d^Relative risk for presence vs absence

### Mutually-adjusted associations

**Fig. 1:**
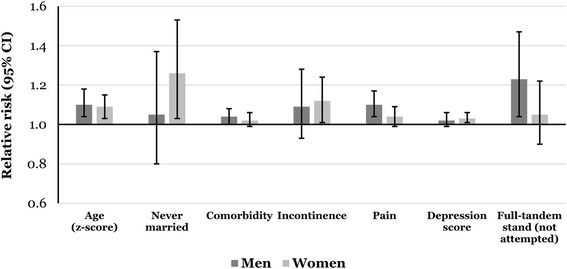
Mutually-adjusted relative risks for incident falls between Waves 4 and 6 among 1515 men and 1783 women aged 60 and over. Relative risks are per unit increase in age, number of comorbidities and depression score (CES-D); compared to those without incontinence; per higher category of pain; compared to those who were married/cohabiting; and compared to those who were able to hold a full-tandem stand for the required time. Relative risks were additionally-adjusted for: previous falls, hearing, vision, frailty status, forced expiratory volume and cognition. Among men, additional adjustments were household wealth, smoking status, physical activity and alcohol consumption frequency; among women, relative risks were additionally adjusted for BMI

Mutually-adjusted relative risks for falling between Waves 4 and 6 among men and women separately are presented in Table 3. Older age was associated (*p* < 0.05) with increased fall risk among men and women. Many risk factors were sex-specific. Among men, experiencing higher levels of pain, poorer balance as indicated by the inability to attempt the full-tandem stand test, and increased morbidity (*p* = 0.052) were associated with increased fall risk in mutually-adjusted analyses. Never being married, incontinence and higher depression scores were associated with increased risk of falling among women only. No other characteristics were associated with higher risk of falls among men or women in mutually-adjusted analyses. Figure 1 shows the relative risks for incident falls according to those factors that were significantly associated with falls risk in men and in women.

**Table 3:** Mutually-adjusted relative risks for incident falls between Waves 4 and 6 among 1515 men and 1783 women aged 60 and over

Characteristic	Men		Women	
	Relative risk (95% CI)	*P*-value	Relative risk (95% CI)	*P*-value
Age (z-score)^a^	1.10 (1.04,1.18)	0.002	1.09 (1.03,1.15)	0.003
Marital status^d^				
Married/cohabiting	1.00		1.00	
Divorced/widowed/separated	1.09 (0.93,1.27)	0.558	1.05 (0.94,1.17)	0.072
Never married	1.05 (0.80,1.37)		1.26 (1.03,1.53)	
Household wealth^b^	1.04 (0.99,1.09)	0.086	–	–
BMI (z-score)^a^	–	–	1.02 (0.97,1.07)	0.411
Smoking status^d^				
Never	1.00			
Ex	1.06 (0.93,1.22)	0.374	–	–
Current	1.16 (0.94,1.44)			
Physical activity^b^	0.98 (0.90,1.07)	0.685	–	–
Alcohol consumption frequency^b^	0.96 (0.92,1.01)	0.133	–	–
Number of comorbidities^a^	1.04 (1.00,1.08)	0.052	1.02 (0.99,1.06)	0.214
Hearing^b^	1.01 (0.96,1.07)	0.700	1.00 (0.95,1.05)	0.981
Vision^b^	1.05 (0.99,1.11)	0.136	1.04 (0.98,1.09)	0.174
Incontinence^c^	1.09 (0.93,1.28)	0.263	1.12 (1.00,1.24)	0.042
Pain^b^	1.10 (1.04,1.17)	0.001	1.04 (0.99,1.09)	0.128
Depression score (CES-D)^a^	1.02 (0.99,1.06)	0.238	1.03 (1.01,1.06)	0.010
Full-tandem stand^d^				
≥ 10 s if aged ≥70/≥30 s if aged < 70	1.00		1.00	
< 10 s if aged ≥70/< 30 s if aged < 70	1.13 (0.97,1.32)	0.039	1.09 (0.96,1.22)	0.407
Not attempted	1.23 (1.04,1.47)		1.05 (0.90,1.22)	
Frailty status^d^				
Not frail	1.00	0.661	1.00	0.706
Pre-frail	1.06 (0.92,1.23)		0.98 (0.88,1.10)	
Frail	1.10 (0.87,1.39)		0.93 (0.78,1.11)	
Forced expiratory volume (z-score)^a^	1.00 (0.94,1.07)	0.931	1.00 (0.95,1.06)	0.997
Cognition (z-score)^a^	1.00 (0.94,1.07)	0.900	1.00 (0.95,1.05)	0.937

Poisson regression models with robust variance estimation were used to yield relative risks

Analyses were adjusted for previous falls before Wave 4

^a^Relative risk per unit increase

^b^Relative risk per higher category

^c^Relative risk for presence vs absence

^d^*P*-value for difference in falls risk between categories

Using the pooled sample of both men and women, we checked whether the associations between comorbidity, pain, incontinence, ability to perform a full-tandem stand, marital status and depressive symptoms, in relation to falls risk, varied significantly between the sexes. In analyses adjusted for the other variables shown in Table 3, the relationships between comorbidity, pain and ability to perform a full-tandem stand and risk of having an incident fall differed significantly between the sexes (*p* values for interaction terms were 0.017, 0.003 and 0.001 respectively); the relationships between marital status, incontinence and depressive symptoms and risk of having an incident fall were not significantly different (*p* values for interaction terms were 0.406, 0.335 and 0.088 respectively).

## Discussion

In this prospective study of 3298 men and women aged 60 or over, we examined the relationship between a wide range of characteristics (sociodemographic, lifestyle, clinical, cognitive and physical) and risk of incident falls over a 4-year period. Of the 17 characteristics examined, only older age was associated with increased risk of falls in both men and women in multivariable-adjusted analyses. Some risk factors were predictive of incident falls in one sex but not in the other. Increased comorbidity (*p* = 0.052), higher levels of pain and poorer balance were associated with a raised risk of falls in men, and being more depressed, incontinence and never having been married were associated with a raised risk in women.

Our finding that higher levels of pain was associated with a significantly greater risk of having an incident fall in men than in women is consistent with recent findings in the Swedish National Study on Aging and Care [27]. In that prospective study, several measures of pain (having daily pain, or pain that affected daily activities, or was at least mild to moderate) were linked with an increased risk of having a fall that required outpatient or inpatient care in men, but not in women [27]. In our study, as in the Swedish study, women were more likely to report higher levels of pain than men. There is considerable evidence, largely from studies using retrospective data on falls, but also from some studies with prospective data, that the presence of pain, and particularly more severe pain, is associated with greater likelihood of falls [28, 29]. With the exception of the study by Welmer et al. [27] we are not aware of any other prospective studies that have reported a sex difference in the relationship between pain and risk of falls. One possible explanation for the sex difference observed in the current study and in that by Welmer et al. may relate to the way women tend to view pain. Welmer et al. suggest that because women tend to be more fearful of pain than men [30], they may behave more carefully when in pain than men do, perhaps becoming less active. Our finding could also be explained if men with pain are less likely than women with pain to use assistive devices when moving around which would affect their risk of falls. Women are more likely than men to develop problems with mobility in later life [31], but while there is evidence that they are more likely than men to use mobility aids [32], a recent study of the use of canes for mobility suggests that women who report poor health or balance may be less likely to use canes than men [33]. We had no information on sites of pain so were unable to explore whether sex differences in this might account for the observation that pain increased risk of incident falls in men but not women.

Several previous cross-sectional studies have reported associations between extent of comorbidity and likelihood of falls [14, 18, 34]. One limitation of these studies is the lack of adjustment for depressive symptoms, physical function and pain, all of which could potentially confound the association between comorbidity and falls. Here, after adjustment for these and other factors, we found a borderline significant association (*p* = 0.052) between greater number of comorbidities and increased risk of incident falls in men. Pooled analysis showed that this association differed significantly by sex, although it is worth noting that the size of the effect was similar in men and women. To our knowledge, no previous prospective study has reported that the relationship between comorbidity and incident falls varies by sex. One explanation for our findings might be that depressive symptoms and pain, both more common in women, play a greater confounding role in the comorbidity-fall relationship in women than in men.

Several previous studies, based either on women only or on pooled samples of both sexes, have found that urinary incontinence is associated with an increased risk of falls, but whether this risk is similar in men and women has been unclear [35]. In the current study, urinary incontinence was associated with increased risk of incident falls in both sexes in minimally-adjusted analyses, however after adjustment for other risk factors this association was significant in women only (*p* = 0.042). The size of the effect in each sex was similar. Nearly three times as many women as men reported having ‘lost any amount of urine beyond their control’ in the last year. The low prevalence of incontinence in men may explain the weaker association with falls risk in multivariable analysis.

A meta-analysis of estimates from 25 prospective studies found a consistent link between higher levels of depressive symptoms and increased risk of falls [15]. Here too, we found that in both men and women, being more depressed at baseline was associated with increased risk of incident falls. After adjustment for other risk factors, this association only remained significant in women, but effect sizes in men and women were similar, and in the pooled sample, we found no evidence that the relationship between depressive symptoms and risk of falls varied significantly by sex.

The ability to maintain a stable posture relies on integrated feedback from sensory, motor and musculoskeletal systems. In the current study, poorer postural balance, as measured by inability to attempt a full-tandem stand, was associated with an increased risk of incident falls among men only after adjusting for vision, hearing, Fried frailty status, cognitive ability and a range of other risk factors. The balance assessment involved participants completing up to three stands, a side-by-side (stand with feet together, side by side); a semi-tandem (stand with the side of the heel of 1 foot touching the big toe of the other foot); and a full-tandem (stand with the heel of 1 foot in front of and touching the toes of the other foot). Only those who successfully held the side-by-side and semi-tandem stands for the required time were invited to attempt the full-tandem stand. We found that the association between the full-tandem stand and fall risk differed significantly by sex. In a study based on a subsample of the Longitudinal Aging Study Amsterdam, inability to hold the full-tandem stand for the required time was associated with increased risk of recurrent falls, as was greater postural sway, but there was no evidence that these associations differed by sex [36].

Our study had several strengths, in particular the fact that ELSA is representative of the English population aged 60 and over living at home, the large size of the sample, and the availability of information on a range of potential risk factors. Many studies of risk factors for falls are cross-sectional and use retrospective data on falls. The prospective design of the current study and the fact that we controlled for a history of falls in the last year makes it possible to be more confident of the direction of effect in the case of factors such as depressive symptoms or pain that can not only increase risk but also be affected by the occurrence of falls. The study also had some weaknesses. The questionnaire did not define what was meant by a fall. Previous researchers have defined falls in a variety of ways and this makes it harder to compare findings [37]. We did not distinguish between people who had fallen once and those who had recurrent falls. We were able to investigate the role of grip strength which was not associated with falls risk among either sex in mutually-adjusted analyses, but we had no data on lower extremity weakness. Evidence suggests that this may be a more powerful determinant of falls risk than grip strength [17].

## Conclusions

In this prospective study of men and women aged 60 or over, we examined the relationship between a wide range of factors and risk of incident falls. Of the 17 risk factors examined only older age was associated with increased risk of falls in both men and women. Several risk factors were predictive of falls in one sex but not in another, but analysis in the pooled sample showed that of these, only the relationships between pain, balance and comorbidity and risk of incident falls differed significantly by sex. Further research should investigate why pain and comorbidity appear to increase the risk of falls in men more than in women.

## Abbreviations

BMI: Body mass index
CAMCOG-R: Cambridge Cognitive Assessment (Revised)
CES-D: Centre for Epidemiologic Depression Scale
FEV1: Forced expiratory volume in 1 second
RR: Relative risk
SD: Standard deviation
